# Analysis of Neutralization Titers against SARS-CoV-2 in Health-Care Workers Vaccinated with Prime-Boost mRNA–mRNA or Vector–mRNA COVID-19 Vaccines

**DOI:** 10.3390/vaccines10010075

**Published:** 2022-01-04

**Authors:** Christina Sølund, Alexander P. Underwood, Carlota Fernandez-Antunez, Signe Bollerup, Lotte S. Mikkelsen, Signe Lysemose Villadsen, Ulrik Fahnøe, Anni Assing Winckelmann, Shan Feng, Caroline A. Nørløv Vinten, Magnus Illum Dalegaard, Greta Vizgirda, Anna-Louise Sørensen, Santseharay Ramirez, Jens Bukh, Nina Weis

**Affiliations:** 1 Department of Infectious Diseases, Copenhagen University Hospital, DK-2650 Hvidovre, Denmark; Christina.soehoel.soelund.01@regionh.dk (C.S.); carlota.fernandez.antunez@regionh.dk (C.F.-A.); signe.bollerup@regionh.dk (S.B.); lotte.scheibelein.mikkelsen@regionh.dk (L.S.M.); signe.lysemose.villadsen@regionh.dk (S.L.V.); anni.assing.winckelmann@regionh.dk (A.A.W.); shan.feng@regionh.dk (S.F.); caroline.amalie.noerloev.vinten.02@regionh.dk (C.A.N.V.); magnus.illum.dalegaard.01@regionh.dk (M.I.D.); greta.vizgirdaite@regionh.dk (G.V.); anna.louise.soerensen@regionh.dk (A.-L.S.); santseharayra@sund.ku.dk (S.R.); jbukh@sund.ku.dk (J.B.); 2 Copenhagen Hepatitis C Program (CO-HEP), Department of Infectious Diseases, Copenhagen University Hospital, DK-2650 Hvidovre, Denmark; alexander.paul.james.underwood@regionh.dk (A.P.U.); ulrik.fahnoee@regionh.dk (U.F.); 3 CO-HEP, Department of Immunology and Microbiology, Faculty of Health and Medical Sciences, University of Copenhagen, DK-2200 Copenhagen, Denmark; 4 Department of Clinical Medicine, Faculty of Health and Medical Sciences, University of Copenhagen, DK-2200 Copenhagen, Denmark

**Keywords:** SARS-CoV-2, COVID-19, mRNA vaccine, vector vaccine, neutralizing/neutralising antibodies, neutralization/neutralisation

## Abstract

With increasing numbers of vaccine-breakthrough infections worldwide, assessing the immunogenicity of vaccinated health-care workers that are frequently exposed to SARS-CoV-2-infected individuals is important. In this study, neutralization titers against SARS-CoV-2 were assessed one month after completed prime-boost vaccine regimens in health-care workers vaccinated with either mRNA–mRNA (Comirnaty^®^, BioNTech-Pfzier, Mainz, Germany/New York, NY, USA, *n* = 98) or vector-based (Vaxzevria^®^, Oxford-AstraZeneca, Cambridge, UK) followed by mRNA-based (Comirnaty^®^ or Spikevax^®^, Moderna, Cambridge, MA, USA) vaccines (*n* = 16). Vaccine-induced neutralization titers were compared to time-matched, unvaccinated individuals that were infected with SARS-CoV-2 and presented with mild symptoms (*n* = 38). Significantly higher neutralizing titers were found in both the mRNA–mRNA (ID_50_: 2525, IQR: 1667–4313) and vector–mRNA (ID_50_: 4978, IQR: 3364–7508) prime-boost vaccine regimens when compared to SARS-CoV-2 infection (ID_50_: 401, IQR: 271–792) (*p* < 0.0001). However, infection with SARS-CoV-2 induced higher titers when compared to a single dose of Vaxzevria^®^ (*p* = 0.0072). Between mRNA–mRNA and vector–mRNA prime-boost regimens, the vector–mRNA vaccine regimen induced higher neutralization titers (*p* = 0.0054). Demographically, both age and time between vaccination doses were associated with vaccine-induced neutralization titers (*p* = 0.02 and *p* = 0.03, respectively). This warrants further investigation into the optimal time to administer booster vaccination for optimized induction of neutralizing responses. Although anecdotal (*n* = 3), those with exposure to SARS-CoV-2, either before or after vaccination, demonstrated superior neutralizing titers, which is suggestive of further boosting through viral exposure.

## 1. Introduction

Severe acute respiratory syndrome coronavirus 2 (SARS-CoV-2), which causes coronavirus disease 2019 (COVID-19), emerged in China in December 2019, and on 11 March 2020, the World Health Organization (WHO) recognized the outbreak of this virus as a global pandemic [1,2]. Infection with SARS-CoV-2 has a wide range of clinical manifestations, ranging from asymptomatic to mild disease to severe disease resulting in hospitalization and even death. Currently, more than 240 million people have been infected, which has resulted in more than five million deaths world-wide [3]. Arguably, some of the most exposed individuals are those that work in a health care setting, who have to treat those infected with SARS-CoV-2 on a regular basis [4,5,6]. It is therefore essential that these individuals are protected from COVID-19, with reduced potential to transmit SARS-CoV-2 infection to others in the health care setting.

The devastating effect of the pandemic called for rapid development of effective and safe therapeutics and vaccines. Vaccinations represent one of the greatest medical advances of modern civilization, and an effective vaccine is the keystone in preventing morbidity and mortality caused by COVID-19. In December 2020, less than a year after the pandemic was recognized, the BNT162b2 mRNA Comirnaty^®^ (BioNTech-Pfzier, Mainz, Germany/New York, NY, USA)COVID-19 vaccine was conditionally approved under an emergency use approval by the Food and Drug Administration (FDA) and the European Medicines Agency (EMA) for administration to individuals to protect against COVID-19 [7,8]. Shortly after, in January 2021, mRNA-1273 Spikevax^®^ (Moderna, Cambridge, MA, USA) and ChAdOx1 nCoV-19 adenoviral Vaxzevria^®^ (Oxford-AstraZeneca, Cambridge, UK) COVID-19 vaccines were approved [9,10]. The conditional approvals were based on interim analyses from phase 3 randomized double blinded placebo-controlled trials that showed a vaccine efficacy of 70% for Vaxzevria^®^ [11] and 94 and 95% for Spikevax^®^ [12] and Comirnaty^®^ [13], respectively. At the end of December 2020, health-care workers in Denmark were administered the first batch of Comirnaty^®^ COVID-19 vaccines in accordance with guidelines issued by the Danish health authorities [5].

Shortly after, Comirnaty^®^ and Vaxzevria^®^ COVID-19 vaccines were deployed in January 2021, followed by the Spikevax^®^ COVID-19 vaccine in February 2021, in accordance with the Danish vaccination strategy which focused on rapidly reducing hospitalizations, severe outcomes, and preventable deaths from COVID-19. In March 2021, the use of Vaxzevria^®^ was put on hold in Denmark due to a report from EMA concerning increased risk of vaccine-induced immune thrombotic thrombocytopenia (VITT) [14] that, in April 2021, led to withdrawal of this vaccine from the Danish vaccination program [15]. In May 2021, those that had received their first dose of Vaxzevria^®^ were permitted to receive their booster dose with either Comirnaty^®^ or Spikevax^®^, resulting in a completed prime-boost vaccine regimen.

Although the correlates of protection against SARS-CoV-2 infection are not completely defined, protection from viral infection is generally attributed to antibodies (Abs), particularly neutralizing Abs (nAbs), which block the virus from interacting with or entering target cells [16,17]. Recently, it has been shown that nAb titers in plasma represent a correlate of protection from COVID-19 in vaccinated and previously infected individuals [18], suggesting that measurement of neutralizing titers can provide insight into the level of protective immunity established. Assessment of nAbs can be performed in vitro using neutralization assays with either whole or pseudotyped viruses [19,20]. While pseudotyped virus assays are a good tool for assessing neutralization in facilities that are not permitted to work with SARS-CoV-2 isolates, they are limited to assessment of the spike protein alone. In contrast, assays utilizing isolates of SARS-CoV-2 may better represent neutralization as these assays employ infectious culture virus, meaning that antibodies to other proteins (such as the nucleocapsid protein) may also bind, showing potential functionality through neutralization. For this reason, a whole virus isolate-based neutralization assay was used in this study.

In the present study, plasma-derived neutralization titers against a SARS-CoV-2 isolate were cross-sectionally assessed one month post prime-boost vaccination in health-care workers vaccinated with either two doses of Comirnaty^®^ (defined as the mRNA–mRNA group) or a prime dose of Vaxzevria^®^ and a booster dose with either Comirnaty^®^ or Spikevax^®^ (defined as the vector–mRNA group) at Copenhagen University Hospital, Hvidovre, Denmark, or at approved COVID-19 vaccination centers in the Zealand or Capital Regions of Denmark. These neutralization titers were compared to time-matched, unvaccinated individuals presenting with mild COVID-19 symptoms who had tested positive for SARS-CoV-2 infection through diagnostic quantitative reverse transcriptase polymerase chain reaction (RT-qPCR). Further, longitudinal neutralizing titers were assessed against an autologous virus for a single case study of a vaccinated individual that later tested positive for SARS-CoV-2 infection.

This study offers important information regarding the level of plasma-derived virus neutralization following vaccination, in both mRNA–mRNA and vector–mRNA prime-boost vaccine regimens, in comparison to those infected with SARS-CoV-2 presenting with mild symptoms. Additionally, this study provides a case report of boosting of neutralizing titers following SARS-CoV-2 infection after vaccination, which may select for or induce broader nAbs. This work is important for understanding vaccine induced immunity, particularly neutralizing antibody responses in health-care workers.

## 2. Materials and Methods

### 2.1. Study Cohort

The Clinical, Virological and Immunological (CVIC) study is a prospective cohort of individuals vaccinated against COVID-19, individuals that have been infected with SARS-CoV-2 and healthy unvaccinated individuals with no previous history of SARS-CoV-2 infection followed at the Department of Infectious Diseases, Copenhagen University Hospital, Hvidovre, Denmark. Health-care workers, who received their prime vaccination between 27 December 2020 and 6 January 2021 and their booster vaccination between 23 January 2021 and 12 February 2021 against SARS-CoV-2 with Comirnaty^®^, were recruited into the CVIC study. In addition, on 29 January 2021, Vaxzevria^®^ was approved by the EMA [10]. Subsequently, a subgroup of health-care workers, who received their prime vaccination with Vaxzevria^®^ between 18 February 2021 and 10 March 2021 and their booster vaccination with either Comirnaty^®^ or Spikevax^®^ between 5 May 2021 and 8 June 2021, were included into the CVIC study. All vaccinated health-care workers working at the hospital site were included based on: >18 years of age, no previous confirmed SARS-CoV-2 infection from routine RT-qPCR and/or antibody testing and able to read and speak adequate Danish to provide written consent. The included participants were required to report on sex, year of birth, dates of vaccination and type of vaccine. Blood was collected in ethylenediaminetetraacetic acid (EDTA) tubes and processed using Ficoll density grade separation to isolate and store plasma and peripheral blood mononuclear cells (PBMCs) at −80 °C and −150 °C at the following three time points: (i) baseline (pre-vaccination), (ii) one month post prime vaccination (only samples taken for subjects receiving prime vaccination with Vaxzevria^®^ were analyzed; this was due to the withdrawal of the Vaxzevria vaccine from the Danish vaccination program and the unknown future vaccination regimen for this group of participants at the beginning of this study) and (iii) one month post boost vaccination. Only participants who had a blood sample collected at one month post booster vaccination were selected for this study.

To achieve a time-matched comparison of neutralizing titers of vaccinated and infected individuals, individuals within the CVIC cohort who had recovered from their SARS-CoV-2 infection and had a baseline time point collected between 21–42 days after symptom onset were selected for this study. All previously infected individuals included had (i) a confirmed SARS-CoV-2 infection diagnosed through routine diagnostic RT-qPCR, (ii) presented with mild COVID-19 (defined by the non-requirement of hospital admission and therapeutic intervention) and (iii) were not vaccinated against COVID-19. All previously infected individuals included had a baseline sample collected between 15 April 2020 and 1 February 2021. Details of their inclusion criteria have been previously described [21].

### 2.2. Ethics Statement

This study complied with the declaration of Helsinki. All participants received oral and written information and gave written consent before inclusion. The study was approved by the Regional Ethical Committee (H-20025872, approved December 2020) and Data Protection Agency (P-2020-357), respectively. All study data were collected and managed using research electronic data capture (REDCap, Vanderbilt University, Nashville, TN, USA) tools hosted at Copenhagen University Hospital, Hvidovre, Denmark [22].

### 2.3. Serological Screening of Participants

Blood samples taken from all participants before the first vaccination and at one month post booster vaccination were subsequently screened for the presence of SARS-CoV-2 receptor binding domain (RBD) total antibodies using the WANTAI SARS-CoV-2 antibody ELISA (Beijing Wantai, Beijing, China, cat#: 256-WS-1096-96), according to the manufacturer’s instructions. Undiluted and non-heat inactivated plasma was used for this assay. Specimens that gave an absorbance value greater than the cut off value (signal/noise ratio > 1.1) were considered positive as per the manufacturer’s recommendations.

### 2.4. Neutralization Assay

The SARS-CoV-2 isolate used in Vero E6 cell-culture experiments was obtained from an individual presenting with COVID-19 at Copenhagen University Hospital, Hvidovre, Denmark, in April 2020 [23], and the neutralization assay was performed as previously described [21]. All experiments using a SARS-CoV-2 isolate were performed under biosafety conditions in agreement with Danish regulations and with permission from the Danish authorities. The sequence of this SARS-CoV-2 isolate (DK-AHH1) can be found in GenBank (accession number MZ049597) [23] and belongs to the Nextstrain Clade 20C. Neutralization experiments were performed by adding the virus (multiplicity of infection (MOI) of 0.03) to two-fold serially diluted plasma (heat inactivated at 56 °C for 30 min) from vaccinated individuals and individuals with mild COVID-19 (starting at a 1:10 dilution and ranging up to a 1:20,480 dilution) at a 1:1 ratio and incubating at room temperature for 1 h. To determine neutralization, pre-vaccine plasma samples (heat inactivated at 56 °C for 30 min) were included as negative controls. In addition, given that those infected with SARS-CoV-2 did not have a pre-exposure time point, pooled plasma (heat inactivated at 56 °C for 30 min) from five healthy individuals was used as a negative control. A mouse derived SARS-CoV-2 spike neutralizing antibody (Sino Biological, Beijing, China, #40592-MM57, RRID: AB_2857935) was used as a positive control for neutralization. Following 1 h incubation, plasma/virus and antibody/virus complexes were then added to Vero E6 cells (RRID: CVCL_0574) seeded the day before (10^4^ cells/well; Corning white BioCoat^TM^ Poly-D lysine coated plates, Horsham, PA, USA, cat #: 354651) in quadruplicate. After 48 h incubation at 37 °C and 5% CO_2_, the cells were stained as described previously [21], using mouse-derived spike primary (Sino Biological #40592-MM57,RRID: AB_2857935) and GE Healthcare #NA931V (RRID: AB_772210) secondary antibodies. Spots representing virus infected cells were counted using an Immunospot series 5 UV analyzer (Cellular Technologies, Cleveland, OH, USA. Single outliers of quadruplicates were calculated using a modified z-score system, as previously described [21], and were removed from further analysis; thus, a minimum of triplicates was used for all assays. Healthy plasma and virus only controls have been previously compared in this assay and were not found to be different [21]. Therefore, the percentage neutralization was calculated as:

$$\%\;Neutralization=1-\left(\frac{Spot\;count}{Spot\;count\;\left\{Pre-vaccine\;or\;healthy\;controls\right\}}\right)\times 100$$

Any overall neutralization values (average of the triplicates/quadruplicates) that yielded higher than 100% were normalized to 100% and any overall neutralization values that yielded lower than 0% were normalized to 0%.

### 2.5. Longitudinal Autologous Neutralization

During this study, one vaccinated individual (V-67) became infected with SARS-CoV-2. While this individual was infected, a nasal swab was taken four days post symptom onset and the contained SARS-CoV-2 variant propagated in Vero E6 cells. In addition, the swab was submitted for viral sequencing as previously described [23], and the SARS-CoV-2 isolate was found to be an alpha variant (B.1.1.7, Genbank accession OK041529, Nextstrain Clade 20I (Alpha V1)). After growth in cell culture, the virus was re-sequenced and two amino acid substitutions in the spike protein were found (F157L and R682W). The virus was titrated to obtain a 50% tissue culture infectious dose (TCID50) and autologous neutralization against this isolate was performed, as described above, using a MOI of 0.03 [23].

### 2.6. Statistics

Neutralization curves were constructed in GraphPad Prism (GraphPad Prism Software, San Diego, CA, USA, version 9.1.0.22), and the 50% inhibitory dilution (ID_50_) of plasma was calculated using non-linear regression (Log (inhibitor) vs. normalized response (variable slope)). The statistical tests used here include Fisher’s exact test, the Mann–Whitney *U* test for unpaired data and the Wilcoxon matched-pairs signed rank t test for paired data, which were conducted in GraphPad Prism (version 9.1.0.22). The specific statistical test performed is indicated in the text and figure legends. Categorical variables were reported as absolute numbers and relative frequencies, while continuous variables were summarized as mean with standard deviation and median and interquartile range. A multivariate linear regression analysis was conducted in RStudio (RStudio Team (2020) Integrated Development for R. RStudio, PBC, Boston, MA, USA, URL http://www.rstudio.com/ (accessed on 15 November 2021)). In brief, the multiple linear regression analysis was used with the ID_50_ as the outcome and age, sex and time points between vaccinations as the explanatory variables. The beta estimates (β), 95% confidence intervals (95% CI) and *p* values are reported. The assumption of linearity was tested by plotting residuals against continuous variables. Variance homogeneity was tested by plotting fitted values against residuals, and QQ-plots were used to ensure normally distributed residuals. *p*-values below 0.05 (two-sided) were considered statistically significant.

## 3. Results

### 3.1. Participant Characteristics

The CVIC study has, in total, enrolled 131 vaccinated individuals: 109 who were vaccinated with a mRNA–mRNA prime-boost vaccine regimen (Comirnaty^®^) and 22 who received a vector–mRNA prime-boost vaccine regimen, whereby their prime vaccination was with a vector-based vaccine (Vaxzevria^®^) and their booster vaccination was with a mRNA based vaccine (either Comirnaty^®^ or Spikevax^®^). In addition, the CVIC study has enrolled 103 individuals who have had a confirmed SARS-CoV-2 infection resulting in mild COVID-19.

**Figure 1:**
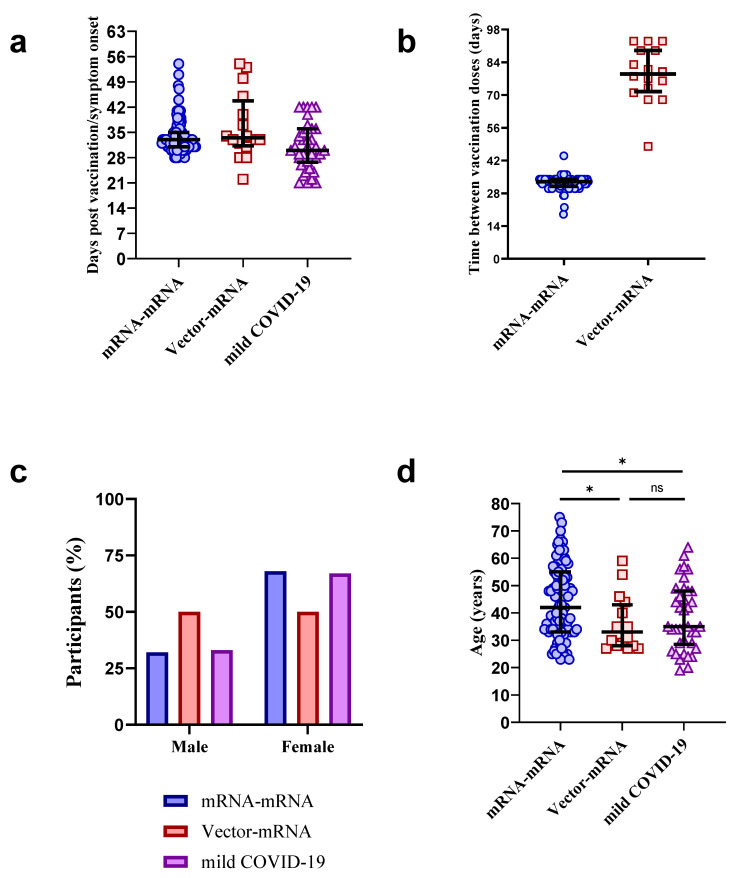
Participant characteristics. Distribution of days post booster COVID-19 vaccination or post symptom onset after SARS-CoV-2 infection (**a**), time between prime and boost vaccination doses (**b**), sex (**c**) and age (**d**) in mRNA–mRNA (blue, *n* = 98), vector–mRNA (red, *n* = 16) and mild COVID-19 (purple, *n* = 38) groups. The mRNA–mRNA group was found to be significantly older than the vector–mRNA and mild COVID-19 groups (* *p* = 0.018 and * *p* = 0.021, respectively, Mann–Whitney *U* tests). ns = not significant. The bold lines represent the median and interquartile range.

Of the 109 participants in the mRNA–mRNA group, 98 (90%) had a blood sample taken at a median of 33 days (Interquartile range (IQR) = 31–35) post booster vaccination and were selected for this study (Figure 1a). The remaining 11 (10%) were excluded due to either withdrawal of participation in the CVIC study (*n* = 5), did not receive their second vaccination on time (*n* = 2) or did not attend their blood collection visit (*n* = 4).

Of the 22 individuals in the vector–mRNA group, 18 (81%) had a blood sample collected at a median of 37 days (IQR = 34–42) post Vaxzevria^®^ vaccination, and a further 16/18 (89%) had a blood sample collected at a median of 34 days (IQR= 32–34) post booster vaccination (Figure 1a). Only those with a blood sample collected after their booster vaccination were selected for this study (*n* = 16). The remainder either withdrew from the CVIC study (*n* = 3) or did not attend their blood collection visit (*n* = 3). Of the 16 individuals included in this study, 15 (94%) received Comirnaty^®^ and 1 (6%) received Spikevax^®^ for their booster vaccination. The mRNA–mRNA group received their booster vaccine at a median of 33 days after their prime vaccine (IQR = 31–34), which was found to be earlier than the vector–mRNA group, who received their booster vaccine at a median of 79 days (IQR= 73–89) after their prime vaccine (Figure 1b).

Of the 103 individuals with mild COVID-19, 38 (37%) had a baseline time point collected approximately 21–42 days post symptom onset (median days post symptom onset = 30 (IQR = 28–36)) and were selected for this study (Figure 1a). Thirteen of these 38 (34%) individuals have had their neutralizing titers previously reported [21]. In the mRNA–mRNA group, 66 (67%) were female, while 8 (50%) and 25 (66%) participants were female in the vector–mRNA group and the mild COVID-19 group, respectively (Figure 1c). The median age in years was 43 (IQR = 33–55), 33 (IQR = 28–41) and 35 (IQR = 29–48) for the mRNA–mRNA group, vector–mRNA group, and mild COVID-19 group, respectively (Figure 1d). It is important to note that both the vector–mRNA and mild COVID-19 groups were found to be significantly younger than the mRNA–mRNA group (*p* = 0.018 and *p* = 0.021, respectively, Mann–Whitney *U* tests). However, no significant age difference was found between the vector–mRNA and mild COVID-19 groups (*p* > 0.05, Mann–Whitney *U* test). A full summary of participant demographics for the mRNA–mRNA group, vector–mRNA group and mild COVID-19 group can be found in Supplementary Tables S1–S3, respectively.

### 3.2. Testing for Prior SARS-CoV-2 Infection in Vaccinated Individuals by WANTAI ELISA

Blood samples taken pre-vaccination and at one month post booster vaccination from the included participants were screened using the WANTAI ELISA to determine any prior exposure to SARS-CoV-2 and evaluate the development of nAbs. Of the mRNA–mRNA participants, 1/98 (1.0%) was found to be positive in their pre-vaccination time point (V-70). In the vector–mRNA group, 1/16 (6.2%) was found to be positive in their pre-vaccination time point (V-116). Both positive individuals reported potential exposure to someone infected with SARS-CoV-2 but had not received a positive PCR result through routine diagnostic testing. Participant V-70 had a negative antibody test performed two months before COVID-19 vaccination, and a PCR test for SARS-CoV-2 was performed seven times with a maximum of a two-week interval between the antibody test and pre-vaccine sampling. For participant V-116, an antibody test was performed five months before COVID-19 vaccination, and eleven PCR tests were conducted with a maximum of a three-week interval between the antibody test and the pre-vaccine sampling. All mild COVID-19 individuals (*n* = 38) were found to test positive. The WANTAI test results for each individual participant can be found in Supplementary Tables S1–S3.

### 3.3. Comparison of Neutralization Titers between mRNA–mRNA, Vector–mRNA and Mild COVID-19 Groups

**Figure 2:**
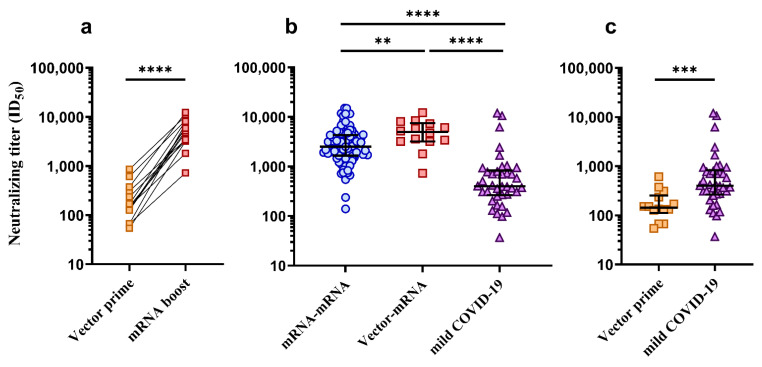
Comparison of neutralizing titers (ID_50_) between mRNA–mRNA and vector–mRNA prime-boost COVID-19 vaccine regimens and SARS-CoV-2 infected individuals presenting with mild COVID-19. (**a**) Comparison of neutralizing titers in the vector–mRNA group (*n* = 15) between the one-month post prime dose (Vector prime, yellow squares) vs. one-month post booster (mRNA boost, red squares). Neutralizing titers at the one-month post boost time point were found to be significantly higher than the one-month post prime time point (**** *p* < 0.0001, Wilcoxon ranked *t* test). (**b**) Comparison of neutralizing titers between the mRNA–mRNA (blue circles, *n* = 96), vector–mRNA (red squares, *n* = 15) and mild COVID-19 (purple triangles, *n* = 38) groups at one month post boost or one month post symptom onset. The vector–mRNA group was found to have significantly higher neutralizing titers when compared to the mRNA–mRNA group (** *p* = 0.0071, Mann–Whitney *U* test). Both the vector–mRNA group and the mRNA–mRNA group were found to have significantly higher neutralizing titers when compared to the mild COVID-19 group (**** *p* < 0.0001, Mann–Whitney *U* tests). (**c**) Comparison of neutralizing titers between the vector–mRNA group (*n* = 15) at one month post prime dose (Vector prime, yellow squares) and the mild COVID-19 group (purple triangles). Those in the mild COVID-19 group were found to have significantly higher neutralizing titers (*** *p* = 0.0003, Mann–Whitney *U* test). The bold lines represent the median and interquartile range.

In the mRNA–mRNA group (*n* = 98), the median ID_50_ at one month post booster vaccination was 2525 (IQR = 1667–4313). In the vector–mRNA group (*n* = 16), the median ID_50_ one month after prime vector-based vaccination (Vaxzevria^®^) was 143 (IQR = 112–254) and the median ID_50_ one month after booster vaccination with an mRNA vaccine was 4978 (IQR = 3364–7508), which, when compared, was found to be a highly significant boost in neutralizing titers (Figure 2a, *p* < 0.0001, Wilcoxon *t* test). In the mild COVID-19 group (*n* = 38), the median ID_50_ was 401 (IQR = 271–792). The two subjects that had tested positive in the WANTAI ELISA prior to receiving their vaccines (V-70 and V-116) showed exceptionally high neutralizing titers (V-70 ID_50_ = 10195 and V-116 ID_50_ = 10446) compared to their respective groups’ median ID_50_ of 2525 (mRNA–mRNA group) and 4978 (vector–mRNA group). On the other hand, it was also found that 3/98 (3%) subjects in the mRNA–mRNA group showed exceptionally low neutralizing titers (V-02 ID_50_ = 101, V-34 ID_50_ = 239 and V-87 ID_50_ = 140) when compared to the respective median ID_50_. Upon follow-up with these individuals, one (V-02) reported recent use of immunosuppressant therapy while the other two (V-34 and V-87) did not report any reason for their low neutralizing titers. To normalize comparisons of neutralizing titers between groups, all individuals with previous SARS-CoV-2 exposure (V-70 and V-116) and with reported immunosuppressive therapy (V-02) were excluded from further analysis. As shown in Figure 2b, comparisons between the mRNA–mRNA group (*n* = 96) and the vector–mRNA group (*n* = 15) at the one-month post booster vaccination time point showed significantly higher neutralizing titers in the vector–mRNA group (*p* = 0.0071, Mann–Whitney *U* test). Furthermore, both groups were found to have significantly higher neutralizing titers compared to the mild COVID-19 group (*n* = 38) (Figure 2b, *p* < 0.0001, Mann–Whitney *U* tests). However, when the mild COVID-19 group (*n* = 38) was compared to the vector–mRNA group at the one-month post prime vector-based vaccination time point (*n* = 15), neutralizing titers were found to be significantly higher in the mild COVID-19 group (Figure 2c, *p* = 0.0003, Mann–Whitney *U* test).

### 3.4. Lower Vaccine-Induced Neutralizing Titers May Be Associated with Age

**Figure 3:**
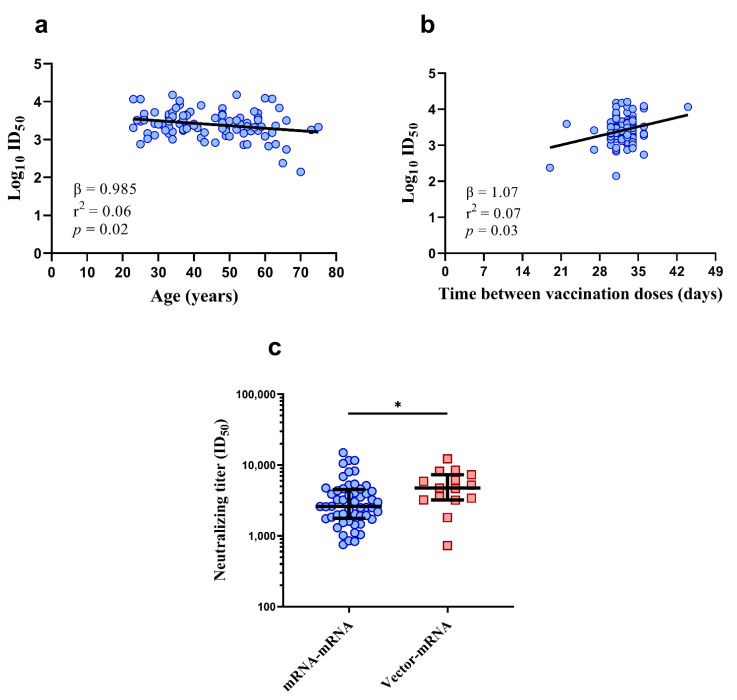
Distribution of neutralizing titers with age (**a**) and time between vaccination doses (**b**) in the mRNA–mRNA group (*n* = 96). Comparison of age-matched neutralizing titers between the mRNA–mRNA (*n* = 52, blue circles) and vector–mRNA (*n* = 15, red squares) groups (**c**). Despite age-matching, neutralization titers remained significantly higher in the vector–mRNA group (* *p* = 0.020, Mann–Whitney *U* test). For (**a**,**b**), the bold lines represent the linear regression. For (**c**), the bold lines represent the median and interquartile range.

To understand if the neutralizing titers elicited were associated with demographic factors, a multivariate linear regression analysis of sex, age, time between vaccinations and time post vaccination was conducted on the mRNA–mRNA group (*n* = 96, V-02 and V-70 were excluded). The vector–mRNA group was excluded from this analysis due to the small sample size (*n* = 15). Since ID_50_ was log transformed to achieve a linear fit, estimates from the regression model show percentage change in ID_50_. As shown in Figure 3a,b, there was an association between higher age and lower neutralization titers, where the ID_50_ fell 1.5% per one-year increase in age (β = 0.985; 95% CI: 0.973–0.997, *p* = 0.02) and an association between neutralizing titers and time between the prime and booster vaccinations with a 7% increase in ID_50_ per day within a range of 19–44 days (β = 1.07; 95% CI: 1.01–1.14; *p* = 0.03). No associations between neutralizing titers were observed for sex (β = 0.85; 95% CI: 0.60–1.20; *p* = 0.36) or for the time post vaccination (β = 0.99; 95% CI: 0.96–1.03; *p* = 0.62).

Given the association between age and vaccine-induced neutralizing titers, and that the mRNA–mRNA group was significantly older than the vector–mRNA group, neutralizing titers were re-compared between these groups using age-matched individuals from the mRNA–mRNA group (individuals 46 years old or younger, *n* = 52, median age = 34 (IQR = 29–38)). Despite accounting for age, the neutralizing titers in the vector–mRNA group at one month post booster vaccination remained significantly higher (Figure 3c, *p* = 0.020, Mann–Whitney *U* test).

### 3.5. A Single Case of Symptomatic SARS-CoV-2 Infection Following mRNA–mRNA (Comirnaty^®^) Vaccination

**Figure 4:**
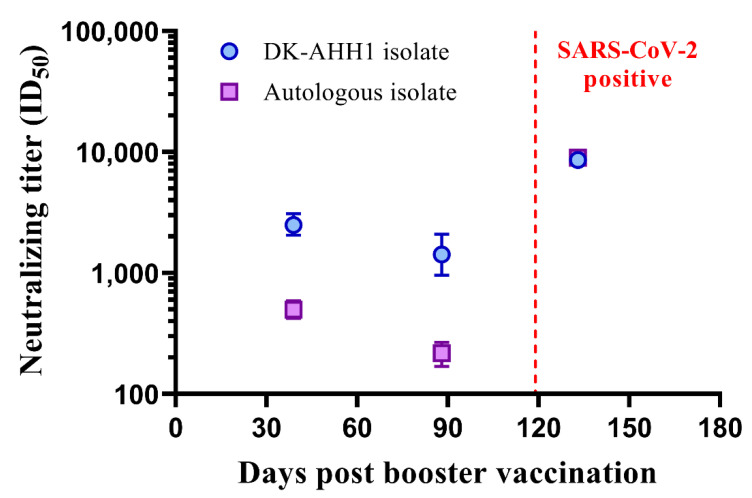
SARS-CoV-2 reinfection in mRNA–mRNA vaccinated individual. The graphs show the longitudinal neutralizing titers for V-67, who was infected with SARS-CoV-2 after completed prime-boost vaccination with a mRNA–mRNA (Comirnaty^®^) regimen, against the DK-AHH1 isolate (blue circles) and the autologous isolate (purple squares), with the infection point marked with a red dotted line (day 119 post booster vaccination).

During this study, one subject (V-67) that received a mRNA–mRNA prime-boost vaccination (Comirnaty^®^) tested positive for SARS-CoV-2 infection by PCR at 119 days post booster vaccination. This subject had reported symptoms of fever, dry cough and blocked nose, which receded after five days. Genomic analysis of SARS-CoV-2 recovered from a nasal swab taken from this subject determined the infecting virus to be Clade 20I (Alpha V1), which propagated in cell culture. In addition to the blood sample taken one month post booster vaccination (39 days), a blood sample had also been taken at 88 days post booster vaccination, which was 31 days prior to the SARS-CoV-2 infection, and at 133 days post booster vaccination, which was 8 days post-symptom onset. Longitudinal examination of neutralizing titers was determined against both the DK-AHH1 isolate of SARS-CoV-2 (i.e., the one used to screen for neutralizing titers for all other subjects) and the autologous isolate (i.e., the one isolated from the subject during infection). As shown in Figure 4, after vaccination, neutralizing titers against DK-AHH1 (mean ID_50_ = 2503 (95% CI: 2058–3091)) were higher than those of the autologous isolate (mean ID_50_ = 498 (95% CI: 421–590)). For both viruses, neutralizing titers were seen to wane at 88 days post booster vaccination (DK-AHH1, mean ID_50_ = 1427 (95% CI: 961–2091); autologous isolate, mean ID_50_ = 217 (95% CI:169–267)). Following infection, there was a large anamnestic response seen in neutralizing titers, with both variants reaching comparable neutralizing titers (DK-AHH1, mean ID_50_ = 8628 (95% CI: 7807–9535); autologous isolate, mean ID_50_ = 8978 (95% CI: 7820–10,225)).

## 4. Discussion

Understanding the level of immunity generated by the COVID-19 vaccines is paramount for health-care workers who are frequently exposed to, and at a high risk of, infection with-SARS-CoV-2 due to their employment. It is also important to compare the level of immunity generated in vaccinated individuals to that of SARS-CoV-2 infected individuals, allowing a deeper understanding of just how immunogenic the COVID-19 vaccinations are. While it has been shown that vaccination of health-care workers and other hospital staff can reduce risk of infection with SARS-CoV-2 and progression of associated disease [24,25], the level of vaccine-induced immunity has not been measured in these studies. Given that nAbs have recently been found to be a correlate of protection from disease [18], the present study assessed neutralizing titers in plasma from vaccinated health-care workers as a means of assessing potential levels of protection. Interestingly, individuals with completed prime-boost vaccine regimens were found to have significantly higher neutralization titers when compared to time-matched individuals who had recovered from mild COVID-19 infection, which has also been reported by others [26]. Remarkably, even after accounting for age differences, those receiving a vector–mRNA prime-boost regimen had significantly higher neutralizing titers than those receiving a mRNA–mRNA prime-boost vaccine regimen, which is also concordant with results found in other studies [27,28,29]. Although neutralizing titers following the prime vaccination in the mRNA–mRNA group were not assessed, it is reassuring to see a largely significant boost in neutralizing titers between prime and boost vaccinations in the vector–mRNA group. Interestingly, however, neutralizing titers in those with mild COVID-19 were significantly higher than those receiving only a prime dose of Vaxzevria^®^, suggesting that a single Vaxzevria^®^ dose is not enough to reach comparable neutralizing titers to that of infection with SARS-CoV-2. Furthermore, this highlights the requirement for prime-boost COVID-19 vaccine regimens to achieve neutralizing titers which surpass that of neutralizing titers induced by SARS-CoV-2 infection alone.

Consistent with findings reported by others [30,31,32], vaccine-induced neutralizing titers were observed to be slightly lower with increasing age, suggesting that age is a significant factor for induction of neutralizing responses. However, it is important to note that the observed effect of age was small in this study, and there were only a few subjects >70 years included. Furthermore, the association of the time between prime and boost vaccinations to neutralizing titers, with higher titers being found in those that had a longer time between their vaccination doses, was largely driven by a small number of participants that were vaccinated outside of the bulk of the participants (around 30 days post prime). However, given that the optimal time to administer booster vaccinations after the prime dose is not known, this finding warrants further investigation into optimization of prime-boost vaccine regimens for optimized induction of neutralizing responses. In rhesus macaques, germinal centers, the site at which B cells mature and expand, have been shown to be active for eight weeks following vaccination [33]. One explanation for the observed result in this study could be that the additional time between prime and booster vaccinations allows for longer maturation of B cells before antigen re-exposure. More mature B cells may have gone through longer rounds of somatic hypermutation to become more affinity-matured to the target antigen. Thus, upon antigen re-encounter, the more mature B cells will be selected with increasing levels of expansion.

During this study, one vaccinated individual became infected with a SARS-CoV-2 variant belonging to Nextstrain Clade 20I (Alpha V1), which allowed a unique opportunity to analyze longitudinal neutralization to heterologous and autologous virus following vaccination. It is important to note that two spike amino acid changes were detected in the autologous isolate following propagation in cell culture. However, given that there was no immune selection pressure, it is unlikely that these amino acid changes would affect the observed neutralization. Interestingly, prior to infection, neutralizing titers to the autologous isolate were observed to be lower when compared to the DK-AHH1 isolate. This may be because the DK-AHH1 isolate expresses a spike protein that more closely resembles that of the spike protein within the COVID-19 vaccines. Despite this, this individual did not progress to develop severe COVID-19 and only presented with mild symptoms for up to five days. After 133 days post booster vaccination, following infection, not only were the neutralizing titers to the DK-AHH1 isolate boosted, but the neutralizing titers to the autologous isolate were now at comparable levels. This could suggest that either vaccine-induced cross-reactive B cells were selected for, which in turn drove an anamnestic response of cross-reactive nAbs, or the infection stimulated de novo B cell responses, which generated infection-specific nAbs, or both. In any case, this provides evidence that, following vaccination, exposure to SARS-CoV-2 can boost neutralizing titers and may select for or induce more cross-neutralizing Abs.

During the serological screening for the participants included in this study, two individuals (V-70 and V-116) were found to test positive for anti-RBD antibodies in their pre-vaccination sample, suggesting that they had been previously asymptomatically infected by SARS-CoV-2. However, despite frequent routine diagnostic testing prior to vaccination, these two individuals did not test positive for SARS-CoV-2 RNA or anti-S Abs. This could suggest that these individuals may have been exposed to SARS-CoV-2 antigen without establishment of replication competent infection, thus leading to stimulation of adaptive immunity. Interestingly, after completion of their vaccine regimen, neutralizing titers in these two individuals were among the highest of their respective groups. This is perhaps not surprising as vaccination in individuals with previous SARS-CoV-2 exposure has been shown to boost neutralization titers significantly more than in those without previous exposure [34,35,36]. Taken together, these data support the notion that the effect of vaccination in those with previous SARS-CoV-2 exposure is likely superior to those without previous exposure, thus suggesting that an additional booster vaccination on top of the current prime-boost vaccine regimens may be beneficial for generating superior nAb responses. Given that the EMA and the FDA have approved second booster vaccination doses for those with at least six months since their previous dose [37,38], these results are quite timely. This result also highlights that previous SARS-CoV-2 infected individuals will benefit from vaccination to enhance neutralization responses.

Within the mRNA–mRNA (Comirnaty^®^) group, three subjects (V-02, V-34 and V-87) were found to have markedly lower neutralizing titers than the rest of the group. Upon follow up, while one subject had reported use of immunosuppressive therapy at the time of inclusion (and was therefore excluded), the two other subjects reported no chronic illness or immunosuppressive treatment that could potentially influence their immunogenicity to the vaccine. One limitation within this study is that vaccine-induced T cell immunity was not measured. Therefore, while these subjects may be lacking in neutralization, it may be possible that they generated a more robust T cell response than others. However, given that nAb responses have recently been identified as a correlate of SARS-CoV-2 immunity [18], the lack of neutralization seen may put these individuals at a higher risk of acquiring more severe COVID-19 if infected with SARS-CoV-2.

## 5. Conclusions

This study supports the use of both the mRNA–mRNA and the vector–mRNA prime-boost vaccine regimens to induce neutralization titers superior to that of SARS-CoV-2 infection. Moreover, this study shows, albeit anecdotally, that additional exposure to SARS-CoV-2, be that before or after vaccination, induces greater neutralization titers than vaccination alone. Lastly, this study warrants the investigation into the optimal time to administer booster vaccinations following the prime dose to optimize immunogenicity.

## Data Availability

The data presented in this study are available on request from the corresponding author.

## Supplementary Materials

The following supporting information can be downloaded at: https://www.mdpi.com/article/10.3390/vaccines10010075/s1, Table S1: Summary of 98 subjects receiving mRNA-mRNA prime-boost vaccination with Comirnaty^®^; Table S2: Summary of 16 subjects receiving prime vaccination with Vaxzevria^®^ and boost vaccination with Comirnaty^®^ and Spikevax^®^; Table S3: Summary of 38 subjects included with mild COVID-19.
