# Supplementary material for: Analysis of Neutralization Titers against SARS-CoV-2 in Health-Care Workers Vaccinated with Prime-Boost mRNA–mRNA or Vector–mRNA COVID-19 Vaccines
Source: Vaccines (Basel). 2022 Jan 4;10(1):75. doi: 10.3390/vaccines10010075 (PMC8780959; doi:10.3390/vaccines10010075)
Supplement: Supplementary file 1 [file vaccines-10-00075-s001.zip › vaccines-1486046-supplementary.pdf]

vaccines

MDPI

Supplementary Table S1: Summary of 98 subjects receiving mRNA-mRNA prime-boost vaccination with Comirnaty® (BioNTech-Pfzier, Mainz, Germany/New York, United States)

Patient ID	Age (years)	Sex	SARS-COV-2 WANTAI ELISA Baseline			SARS-COV-2 WANTAI ELISA 1M post 2nd vaccination			1M post 2nd vaccination ID_{50}	Time between 1. and 2. vaccination (days)	Time between 2. vaccination and blood sample (days)
			OD_{450nm}	Ratio (S/N)	Ab	OD_{450nm}	Ratio (S/N)	Ab			
V-01	75	Male	0,02	0,2	Negative	3,88	168,7	Positive	2114	36	28
V-02	67	Male	0,01	0,1	Negative	3,59	156,3	Positive	101	33	31
V-03	46	Male	0,00	0,0	Negative	4,06	176,6	Positive	3704	34	30
V-04	54	Male	0,01	0,0	Negative	3,99	173,5	Positive	722	31	36
V-05	41	Female	0,03	0,3	Negative	3,91	170,2	Positive	2031	34	32
V-06	58	Female	0,03	0,2	Negative	3,95	172,0	Positive	4941	33	31
V-07	36	Female	0,05	0,4	Negative	3,94	171,6	Positive	2599	27	38
V-08	66	Female	0,05	1,0	Negative	4,00	173,9	Positive	547	36	39
V-09	55	Female	0,01	0,0	Negative	3,87	168,2	Positive	2734	33	33
V-10	33	Female	0,00	0,0	Negative	4,03	175,3	Positive	1920	33	41
V-11	48	Female	0,04	1,0	Negative	4,07	58,0	Positive	6643	34	31
V-12	37	Female	0,00	0,0	Negative	3,96	172,5	Positive	1716	33	32
V-13	49	Female	0,00	0,0	Negative	3,98	173,0	Positive	1259	34	31
V-14	36	Male	0,00	0,0	Negative	3,79	164,8	Positive	8243	33	41
V-15	42	Female	0,00	0,0	Negative	3,86	168,0	Positive	1105	33	41
V-16	38	Male	0,01	0,0	Negative	3,89	169,3	Positive	1978	33	41
V-18	62	Female	0,04	0,1	Negative	3,99	173,4	Positive	11872	33	35
V-19	48	Female	0,00	0,0	Negative	3,93	170,9	Positive	4709	34	30
V-20	52	Female	0,00	0,3	Negative	4,00	173,9	Positive	1840	36	28
V-21	36	Male	0,01	0,0	Negative	4,09	178,1	Positive	10536	36	31
V-23	58	Female	0,00	0,0	Negative	3,94	171,5	Positive	1713	34	34
V-24	49	Female	0,01	0,1	Negative	3,90	169,7	Positive	1926	34	33
V-26	60	Male	0,01	0,0	Negative	3,97	172,8	Positive	1215	33	33
V-27	48	Female	0,10	0,1	Negative	4,02	175,1	Positive	4356	30	35
V-28	60	Female	0,02	0,1	Negative	4,08	177,6	Positive	12316	36	30
V-29	58	Female	0,00	0,9	Negative	3,93	171,0	Positive	3718	34	30
V-30	33	Female	0,00	0,1	Negative	3,99	173,6	Positive	1438	34	31
V-31	49	Male	0,00	0,0	Negative	3,99	173,7	Positive	2653	33	34
V-32	31	Female	0,01	0,0	Negative	3,99	173,8	Positive	2498	33	54
V-33	55	Male	0,01	0,0	Negative	3,83	166,8	Positive	743	33	31
V-34	65	Male	0,04	0,6	Negative	3,91	55,0	Positive	239	19	48
V-35	53	Male	0,01	0,1	Negative	3,92	170,5	Positive	3740	34	32
V-36	33	Female	0,01	0,1	Negative	3,92	170,6	Positive	3872	33	32
V-37	64	Female	0,01	0,1	Negative	3,97	172,8	Positive	6831	31	34
V-38	52	Female	0,01	0,1	Negative	3,91	170,2	Positive	15142	31	35
V-39	53	Female	0,01	0,1	Negative	4,00	174,2	Positive	2523	34	30
V-42	33	Male	0,09	0,1	Negative	3,95	171,7	Positive	4632	32	39
V-43	29	Male	0,01	0,1	Negative	4,02	174,8	Positive	2615	31	40
V-44	62	Male	0,03	0,8	Negative	3,81	165,7	Positive	1519	31	33
V-45	66	Male	0,05	0,1	Negative	3,95	171,9	Positive	3165	31	33
V-46	34	Male	0,03	0,3	Negative	3,98	173,2	Positive	5138	34	31
V-47	73	Male	0,01	0,5	Negative	4,00	173,9	Positive	1809	31	33
V-48	38	Female	0,00	0,3	Negative	4,10	178,2	Positive	4582	32	37
V-49	25	Female	0,05	0,1	Negative	4,06	176,4	Positive	11633	31	40
V-50	35	Female	0,07	0,0	Negative	4,04	175,7	Positive	6872	31	38
V-51	43	Male	0,07	0,5	Negative	4,00	174,1	Positive	1539	32	30
V-52	39	Female	0,04	0,6	Negative	4,11	57,0	Positive	5384	31	35
V-53	61	Female	0,04	0,6	Negative	4,10	56,9	Positive	670	31	35
V-54	49	Female	0,05	0,6	Negative	4,06	56,4	Positive	1680	34	28
V-56	42	Male	0,06	0,9	Negative	4,05	56,3	Positive	8002	31	35
V-57	25	Male	0,05	0,7	Negative	3,90	54,1	Positive	3029	34	31
V-59	26	Male	0,07	1,0	Negative	4,04	56,0	Positive	3530	32	32
V-60	57	Female	0,04	0,6	Negative	3,97	55,2	Positive	5489	31	31
V-61	63	Female	0,04	0,6	Negative	3,96	55,0	Positive	2315	31	32
V-62	34	Female	0,11	1,5	Negative	4,07	58,0	Positive	3928	22	41
V-63	48	Male	0,05	0,7	Negative	4,04	56,0	Positive	2780	31	34
V-64	24	Female	0,04	0,6	Negative	4,19	60,0	Positive	3238	31	39
V-65	40	Female	0,04	0,6	Negative	4,12	57,3	Positive	2577	31	34
V-66	34	Female	0,05	0,6	Negative	4,05	56,3	Positive	14976	32	30
V-67	32	Female	0,07	0,9	Negative	4,06	56,4	Positive	2503	31	32
V-68	34	Female	0,07	0,9	Negative	3,93	54,6	Positive	1609	34	44
V-69	29	Female	0,04	0,6	Negative	4,05	56,3	Positive	1302	32	31
V-70	27	Female	2,64	59,0	Positive	4,05	56,3	Positive	10195	34	30
V-71	29	Female	0,08	1,1	Negative	4,01	55,6	Positive	5222	34	31
V-72	48	Female	0,05	0,7	Negative	4,12	57,2	Positive	4389	34	31
V-73	50	Female	0,06	0,8	Negative	4,15	57,6	Positive	1218	34	29
V-76	37	Female	0,04	1,0	Negative	4,19	58,2	Positive	3210	31	30
V-78	38	Male	0,04	1,0	Negative	4,17	58,0	Positive	4330	33	30
V-79	48	Female	0,08	1,0	Negative	4,03	55,9	Positive	1957	33	32
V-80	54	Female	0,04	1,0	Negative	4,10	57,0	Positive	1455	32	33
V-82	33	Female	0,04	1,0	Negative	4,11	57,0	Positive	2190	31	37
V-83	34	Female	0,04	0,6	Negative	4,04	56,1	Positive	1012	30	33
V-84	27	Female	0,08	1,1	Negative	4,12	57,2	Positive	1464	30	32
V-85	37	Male	0,09	1,3	Negative	4,05	56,3	Positive	4262	33	32
V-86	37	Female	0,05	0,6	Negative	4,04	56,1	Positive	1840	30	34
V-87	70	Male	0,05	0,7	Negative	3,85	53,4	Positive	140	31	33
V-88	63	Female	0,05	0,8	Negative	3,99	55,4	Positive	753	31	30
V-89	34	Female	0,04	0,6	Negative	4,03	55,9	Positive	4052	33	31
V-90	56	Female	0,08	1,0	Negative	4,05	56,2	Positive	1663	33	30
V-91	43	Female	0,05	0,7	Negative	3,99	55,4	Positive	851	31	32
V-92	33	Female	0,04	0,6	Negative	4,11	57,0	Positive	2991	31	38
V-93	50	Female	0,06	0,9	Negative	4,08	58,3	Positive	3079	33	36
V-94	23	Female	0,04	0,6	Negative	4,09	56,8	Positive	11612	44	28
V-95	26	Female	0,05	0,6	Negative	4,05	56,3	Positive	4753	34	33
V-96	26	Female	0,06	0,8	Negative	3,92	54,5	Positive	3241	30	31
V-97	52	Female	0,08	1,1	Negative	4,04	56,1	Positive	3167	30	31
V-98	32	Male	0,04	1,0	Negative	4,05	56,2	Positive	1748	30	34
V-99	57	Male	0,06	0,8	Negative	4,05	56,2	Positive	2103	30	35
V-100	55	Female	0,05	0,7	Negative	4,28	59,4	Positive	2210	31	33
V-101	25	Male	0,04	1,0	Negative	4,08	56,7	Positive	754	27	30
V-102	58	Female	0,04	1,0	Negative	4,16	57,8	Positive	3314	36	51
V-103	40	Male	0,04	1,0	Negative	4,17	107,0	Positive	2334	33	47
V-104	59	Male	0,04	1,0	Negative	4,10	104,9	Positive	2182	30	31
V-105	30	Female	0,04	1,0	Negative	4,10	105,2	Positive	2526	32	32
V-106	27	Female	0,04	1,0	Negative	4,00	102,2	Positive	1041	34	39
V-107	38	Female	0,04	1,0	Negative	4,00	101,5	Positive	1940	33	33
V-108	46	Male	0,04	1,0	Negative	4,00	101,0	Positive	832	34	34
V-109	23	Female	0,04	1,0	Negative	4,00	103,5	Positive	2038	36	32
Mean (SD)	*44.3 (13.3)*	*Male 32 (33%)*			*Negative 97 (99%)*				*3518.5 (3145.1)*	*32.3 (2.8)*	*34.0 (4.8)*
Median (IQR)	*43 (33 - 55)*	*Female 66 (67%)*			*Positive 1 (1%)*				*2525 (1667.3 - 4313.0)*	*33 (31 - 34)*	*33 (31 - 35)*

Ab= antibody, OD= optical density, ELISA= enzyme-linked immunosorbent assay, S/N= signal/noise, ID50= 50% inhibitory dilution, SD=standard deviation, IQR= interquartile range, 1M = 1 month.

Supplementary Table S2: Summary of 16 subjects receiving prime vaccination with Vaxzevria® and boost vaccination with Comirnaty® (BioNTech-Pfzier, Mainz, Germany/New York, United States) or Spikevax® (Moderna, Cambridge, United States)

Patient ID	Age (years)	Sex	SARS-COV-2 WANTAI ELISA Baseline			SARS-COV-2 WANTAI ELISA 1M post 2nd vaccination			1M post 1st vaccination ID_{50}	1M post 2nd vaccination ID_{50}	Time between 1. and 2. vaccination (days)	Time between 2. vaccination and blood sample (days)	Booster vaccine
			$OD_{450nm-620nm}$	Ratio (S/N)	Ab	$OD_{450nm-620nm}$	Ratio	Ab					
V-110	29	Female	0,04	1,0	Negative	3,4	87,9	Positive	153	3589	73	53	BNT162b2/Comirnaty®
V-111	54	Female	0,04	1,0	Negative	3,6	91,1	Positive	134	6102	93	33	Moderna mRNA-1273/Spikevax®
V-112	27	Male	0,04	1,0	Negative	0,4	10,9	Positive	152	1811	68	50	BNT162b2/Comirnaty®
V-115	27	Female	0,04	1,0	Negative	2,5	64,8	Positive	133	4744	89	34	BNT162b2/Comirnaty®
V-116	28	Female	0,12	2,7	Positive	3,8	98,5	Positive	862	10466	80	32	BNT162b2/Comirnaty®
V-117	40	Male	0,04	1,0	Negative	3,4	86,2	Positive	235	3228	83	28	BNT162b2/Comirnaty®
V-118	28	Male	0,04	1,0	Negative	3,8	98,7	Positive	609	8498	48	37	BNT162b2/Comirnaty®
V-119	30	Male	0,04	1,0	Negative	1,6	40,1	Positive	311	3409	71	45	BNT162b2/Comirnaty®
V-120	59	Male	0,04	1,0	Negative	3,7	94,9	Positive	127	5211	78	34	BNT162b2/Comirnaty®
V-123	31	Female	0,04	1,0	Negative	1,6	40,7	Positive	67	7297	89	31	BNT162b2/Comirnaty®
V-124	27	Male	0,05	1,1	Negative	1,4	36,8	Positive	21	4651	76	54	BNT162b2/Comirnaty®
V-126	35	Male	0,04	1,0	Negative	1,4	36,7	Positive	67	730	68	40	BNT162b2/Comirnaty®
V-127	35	Male	0,04	1,0	Negative	3,3	85,5	Positive	54	3192	93	28	BNT162b2/Comirnaty®
V-128	44	Female	0,04	1,0	Negative	2,6	68,1	Positive	132	5895	81	33	BNT162b2/Comirnaty®
V-129	35	Female	0,05	1,1	Negative	4,1	106,4	Positive	375	12257	77	33	BNT162b2/Comirnaty®
V-130	46	Female	0,04	1,0	Negative	1,4	36,2	Positive	169	8141	93	22	BNT162b2/Comirnaty®
Mean (SD)	*36 (10.06)*	*Male 8 (50%)*			*Negative 15 (94%)*				*225.1 (224.0)*	*5576.3 (3121.8)*	*78.75 (11.9)*	*36.69 (9.3)*	
Median (IQR)	*33 (28 - 41)*	*Female 8 (50%)*			*Positive 1 (6%)*				*143 (112 - 254)*	*4977.5 (3363.8 - 7508.0)*	*79 (73 - 89)*	*34 (32 - 41)*	

Ab= antibody, OD= optical density, ELISA= enzyme-linked immunosorbent assay, S/N= signal/noise, ID50= 50% inhibitory dilution, SD=standard deviation, IQR= interquartile range, 1M = 1 month.

Supplementary Table S3: Summary of 38 subjects included with mild COVID-19

Patient ID	Age (years)	Sex	SARS-COV-2 WANTAI ELISA Baseline			Baseline timepoint (days post symptom onset)	ID_{50} at baseline timepoint
			$OD_{450nm-620nm}$	Ratio (S/N)	Ab		
M-01	25	Female	2,84	15,0	Positive	23	395
M-03	29	Female	1,51	8,0	Positive	30	252
M-04	56	Male	3,09	16,3	Positive	28	12013
M-10	26	Female	3,13	16,4	Positive	29	1045
M-11	20	Male	3,05	16,0	Positive	42	166
M-12	29	Male	3,00	15,8	Positive	33	699
M-13	35	Male	2,92	15,4	Positive	42	366
M-16	49	Female	3,20	16,6	Positive	37	2435
M-17	42	Male	3,06	16,1	Positive	24	937
M-19	64	Female	0,76	4,0	Positive	26	309
M-30	44	Female	2,89	15,2	Positive	27	10634
M-32	56	Female	0,52	2,7	Positive	28	112
M-34	27	Female	3,04	16,0	Positive	25	310
M-38	33	Female	1,45	7,6	Positive	29	406
M-47	34	Male	0,69	3,7	Positive	42	158
M-57	34	Female	3,10	16,3	Positive	42	797
M-65	42	Male	3,92	100,4	Positive	35	1001
M-66	53	Female	4,13	105,9	Positive	36	951
M-67	23	Male	3,88	99,4	Positive	40	118
M-68	24	Male	3,65	93,6	Positive	42	583
M-69	48	Female	1,04	26,8	Positive	33	98
M-73	33	Female	3,36	86,1	Positive	22	705
M-77	45	Male	4,05	103,9	Positive	33	6254
M-78	30	Female	3,88	99,6	Positive	28	269
M-79	44	Male	2,84	72,7	Positive	29	203
M-80	49	Female	3,25	83,4	Positive	21	276
M-88	25	Female	3,40	48,5	Positive	31	709
M-90	34	Female	1,68	37,6	Positive	36	1679
M-91	41	Male	1,41	20,2	Positive	24	394
M-92	19	Female	0,38	5,4	Positive	29	37
M-93	46	Male	1,59	22,7	Positive	21	130
M-94	48	Female	2,97	42,4	Positive	34	313
M-95	35	Female	3,03	43,2	Positive	36	357
M-96	24	Female	3,85	55,0	Positive	31	418
M-97	35	Female	3,05	43,5	Positive	28	376
M-98	35	Female	4,07	58,1	Positive	30	777
M-100	57	Female	3,81	54,4	Positive	31	509
M-102	61	Female	3,64	52,0	Positive	21	737
Mean (SD)	*38.5 (11.9)*	*Female 26 (67%)*				*31.1 (6.3)*	*1229.4 (2594.3)*
Median (IQR)	*30 (28 - 36)*	*Male 13 (33%)*				*30 (28 - 36)*	*395 (261 - 787)*

Ab= antibody, OD= optical density, ELISA= enzyme-linked immunosorbent assay, S/N= signal/noise, ID50= 50% inhibitory dilution, SD=standard deviation, IQR= interquartile range.
